# Green tea and coffee consumption and risk of kidney cancer in Japanese adults

**DOI:** 10.1038/s41598-022-24090-z

**Published:** 2022-11-24

**Authors:** Yichi Chen, Sarah K. Abe, Manami Inoue, Taiki Yamaji, Motoki Iwasaki, Shuhei Nomura, Masahiro Hashizume, Shoichiro Tsugane, Norie Sawada, the JPHC Study Group, N. Sawada, S. Tsugane, M. Iwasaki, M. Inoue, T. Yamaji, R. Katagiri, Y. Miyamoto, H. Ihira, S. K. Abe, S. Tanaka, T. moriya, T. Minamizono, Y. Shirai, H. Kuniyoshi, T. Yoshimi, H. Sonoda, T. Tagami, T. Ando, T. Kimura, Y. Kokubo, K. Yamagishi, T. Mizoue, K. Nakamura, R. Takachi, J. Ishihara, H. Iso, T. Kitamura, I. Saito, N. Yasuda, M. Mimura, K. Sakata, M. Noda, A. Goto, H. Yatsuya, M. Mimura, T. Hanaoka, A. Hidaka, S. Sasazuki, H. Charvat, T. Shimazu, S. Budhathoki, M. Muto, T. Imatoh, J. Ogata, S. Baba, T. Mannami, A. Okayama, K. Miyakawa, F. Saito, A. Koizumi, Y. Sano, I. Hashimoto, T. Ikuta, Y. Tanaba, H. Sato, Y. Roppongi, T. Takashima, H. Suzuki, T. Sugie, Y. Miyajima, N. Suzuki, S. Nagasawa, Y. Furusugi, N. Nagai, Y. Ito, S. Komatsu, H. Sanada, Y. Hatayama, F. Kobayashi, H. Uchino, Y. Shirai, T. Kondo, R. Sasaki, Y. Watanabe, Y. Miyagawa, Y. Kobayashi, M. Machida, K. Kobayashi, M. Tsukada, Y. Kishimoto, E. Takara, T. Fukuyama, M. Kinjo, M. Irei, H. Sakiyama, H. Sakiyama, K. Imoto, H. Yazawa, T. Seo, A. Seiko, F. Ito, F. Shoji, R. Saito, A. Murata, K. Minato, K. Motegi, T. Fujieda, S. Yamato, K. Matsui, T. Abe, M. Katagiri, M. Suzuki, M. Doi, A. Terao, Y. Ishikawa, H. Sueta, H. Doi, M. Urata, N. Okamoto, F. Ide, H. Goto, R. Fujita, Y. Sou, H. Sakiyama, N. Onga, H. Takaesu, M. Uehara, T. Nakasone, M. Yamakawa, Y. Miyasato, F. Horii, I. Asano, H. Yamaguchi, K. Aoki, S. Maruyama, M. Ichii, M. Takano, Y. Tsubono, K. Suzuki, Y. Honda, S. Sakurai, N. Tsuchiya, M. Kabuto, M. Yamaguchi, Y. Matsumura, S. Sasaki, S. Watanabe, M. Akabane, T. Kadowaki, Y. Takashima, Y. Yoshida, S. Matsushima, S. Natsukawa, H. Sugimura, S. Tominaga, M. Iida, W. Ajiki, A. Ioka, S. Sato, M. Konishi, K. Okada, T. Sobue, Y. Kawaguchi, N. Hamajima, S. Akiba, T. Isobe, Y. Sato, T. Sobue, H. Shimizu, S. Kono, T. Sobue, E. Maruyama

**Affiliations:** 1 Division of Health Medical Intelligence, Human Genome Center, The Institute of Medical Science, The University of Tokyo, Tokyo, Japan; 2 Division of Prevention, National Cancer Center Institute for Cancer Control, 5-1-1 Tsukiji, Chuo-Ku, Tokyo, 104-0045 Japan; 3 Division of Cohort Research, National Cancer Center Institute for Cancer Control, Tokyo, Japan; 4 Division of Epidemiology, National Cancer Center Institute for Cancer Control, Tokyo, Japan; 5 Department of Health Policy and Management, School of Medicine, Keio University, Tokyo, Japan; 6 Tokyo Foundation for Policy Research, Tokyo, Japan; 7 National Institute of Health and Nutrition, National Institutes of Biomedical Innovation, Health and Nutrition, Tokyo, Japan; 8 National Cancer Center, Tokyo, Japan; 9 Iwate Prefectural, Ninohe Public Health Center, Ninohe, Iwate Japan; 10 Akita Prefectural Yokote Public Health Center, Akita, Japan; 11 Nagano Prefectural Saku Public Health Center, Saku, Nagano Japan; 12 Okinawa Prefectural Chubu Public Health Center, Uruma, Okinawa Japan; 13 Ibaraki Prefectural Chuo Public Health Center, Ibaraki, Japan; 14 Niigata Prefectural Nagaoka Public Health Center, Niigata, Japan; 15 Kochi Prefectural Chuo-Higashi Public Health Center, Kochi, Japan; 16 Nagasaki Prefectural Kamigoto Public Health Center, Nagasaki, Japan; 17 Okinawa Prefectural Miyako Public Health Center, Miyakojima, Okinawa Japan; 18 National Cerebral and Cardiovascular Center, Osaka, Japan; 19 University of Tsukuba, Tsukuba, Ibaraki Japan; 20 National Center for Global Health and Medicine, Tokyo, Japan; 21 Niigata University, Niigata, Japan; 22 Nara Women’s University, Nara, Japan; 23 Azabu University, Kanagawa, Japan; 24 Osaka University, Suita, Osaka Japan; 25 Oita University, Oita, Japan; 26 Kochi University, Kochi, Japan; 27 Keio University, Tokyo, Japan; 28 Iwate Medical University, Iwate, Japan; 29 Saitama Medical University, Saitama, Japan; 30 Yokohama City University, Yokohama, Kanagawa Japan; 31 Nagoya University, Nagoya, Aichi Japan; 32 Katsushika Public Health Center, Tokyo, Japan; 33 Ibaraki Prefectural Mito Public Health Center, Ibaraki, Japan; 34 Niigata Prefectural Kashiwazaki and Nagaoka Public Health Center, Nagaoka, Niigata Japan; 35 Osaka Prefectural Suita Public Health Center, Suita, Osaka Japan; 36 Tohoku University, Sendai, Miyagi Japan; 37 Research Institute for Brain and Blood Vessels Akita, Akita, Japan; 38 National Institute for Environmental Studies, Tsukuba, Ibaraki Japan; 39 National Institute of Health and Nutrition, Tokyo, Japan; 40 Tokyo University of Agriculture, Tokyo, Japan; 41 The University of Tokyo, Tokyo, Japan; 42 Kyorin University, Tokyo, Japan; 43 Saku General Hospital, Saku, Nagano Japan; 44 Hamamatsu University School of Medicine, Hamamatsu, Shizuoka Japan; 45 Aichi Cancer Center, Nagoya, Aichi Japan; 46 Osaka Medical Center for Cancer and Cardiovascular Disease, Osaka, Japan; 47 Chiba Prefectural Institute of Public Health, Chiba, Japan; 48 Ehime University, Matsuyama, Ehime Japan; 49 Osaka University, Suita, Japan; 50 Tokyo Medical and Dental University, Tokyo, Japan; 51 Kagoshima University, Kagoshima, Japan; 52 Tokyo Gakugei University, Tokyo, Japan; 53 Sakihae Institute, Gifu, Japan; 54 Kyushu University, Fukuoka, Japan; 55 Kobe University, Hyogo, Japan

**Keywords:** Cancer epidemiology, Epidemiology, Risk factors

## Abstract

The study aimed to evaluate the association between green tea and coffee consumption and the risk of kidney cancer using data from a large prospective cohort study in Japan (the Japan Public Health Center-based Prospective Study: JPHC Study). A total of 102,463 participants aged 40–69 were followed during 1,916,421 person-years (mean follow-up period, 19 years). A total of 286 cases of kidney cancer (199 in men, 87 in women) were identified. Cox proportional hazards regression models were used to estimate hazard ratios (HRs) and 95% confidence intervals (95% CIs) while adjusting for potential confounders. No statistically significant association between green tea intake and kidney cancer risk was found in the total population. Among women who consumed more than five cups of green tea per day, a statistically significant decreased risk was shown with a HR of 0.45 (95% CI: 0.23–0.89), compared to women who rarely consumed green tea. For coffee consumption, the association of kidney cancer risk was not statistically significant. This large prospective cohort study indicated green tea intake may be inversely associated with kidney cancer risk in Japanese adults, particularly in Japanese women.

## Introduction

Kidney cancer is the sixth most frequent cancer in men and the 10th in women, representing 5% and 3% of all newly diagnosed cases, respectively^1^. Globally, kidney cancer accounts for more than 2% of cancer burden^2^. And the global incidence rate of kidney cancer has been consistently increasing for the past decades^3^. Most cases of kidney cancer predominantly arise in the renal parenchyma, also known as renal cell carcinoma (RCC)^1,3^. The most important risk factors related to kidney cancer are age and gender^1^. And there appears a ratio of men to women from 1.5:1 to 2.0:1 with men predominance^1^. Only a few modifiable risk factors have been established for potential preventative or detrimental influence on kidney cancer incidence^4^. Cigarette smoking and obesity have been reported to be positively correlated with kidney cancer risk^1^. For dietary factors, recent studies have demonstrated the beneficial effects of fruit and vegetable consumption and moderate alcohol intake against kidney cancer^5–9^.

There is a pressing need to identify additional contributing risk factors for kidney cancer incidence, particularly in regard to dietary risk factors. Tea and coffee are the most commonly consumed beverage worldwide aside from water^10^. Green tea, particularly, is a popular beverage consumed in Japan, China, and some countries in North Africa and the Middle East^10^. The protective roles of green tea and coffee on health promotion and cancer prevention have attracted great attention, as established preventive strategies against cancer are limited^11–15^.

Epidemiological studies have investigated the effects of green tea and coffee drinking in relationship to kidney cancer incidence^16–20^. For coffee consumption, studies have suggested an inverse relationship between coffee consumption and kidney cancer risk^16–18^. Limited studies focused on the relationship between green tea and the risk of kidney cancer incidence^20,21^. Most studies involved tea consumption in general, and were mainly in Caucasian populations in Europe or America, with no conclusive results^19–22^.The evidence was mostly reported by case–control studies which largely suffered from recall bias and sampling bias, lacking results involving Asian population. Green tea is predominantly consumed in Asian population compared to rest of the world^10^. Studies that failed to include data from Asia would result in uncertainty of the effects of green tea consumption against kidney cancer incidence. No previous study was conducted in a large Asian cohort to evaluate the association between green tea consumption and the risk of kidney cancer development.

We aimed to evaluate the association between green tea and coffee consumption and the risk of kidney cancer using data from a large prospective cohort study (the Japan Public Health Center-based Prospective Study: JPHC Study) in Japan.

## Methods and materials

### Study design

The details of the JPHC Study have been reported in previously published papers^23–25^. The study includes two cohorts: Cohort I established in 1990 and Cohort II in 1993. Data from the baseline survey was used in this study, with response rates of 79% in Cohort I and 84% in Cohort II. A total of 140,420 individuals (68,722 men and 71,698 women) aged 40 to 69 years in 11 prefectural public health center areas (PHC) were initially identified. Exclusion criteria for this study included participants from Katsushika-City due to lack of cancer data (n = 7097), ineligibility of non-Japanese nationality, no exist from start, inadequate age, duplicated identification number or refusal for follow-up (n = 284), incorrect response time (n = 113), no response for baseline survey (n = 26,740), incorrect person-years (n = 485), missing information on green tea and coffee consumption (n = 1865) and on body mass index and smoking status (n = 1373). Finally, this resulted in the inclusion of 102,463 participants (48,647 men and 53,816 women). Self-administered questionnaires including items on demographic characteristics, medical history, diet, physical activity, and other lifestyle habits were distributed to all cohort subjects at baseline. All research participants were informed of the study objectives at their enrollment. Informed consent was obtained when participants completed the baseline questionnaire. Incomplete answers were supplemented by telephone interviews.

### Follow up

Participants were followed from the time they responded to the baseline survey through December 31, 2013 (December 31, 2012 for Suita-City). Person-years of observation were established as the time from the date of response to the date of kidney cancer diagnosis, the date of moving-out from the study area, or the end date of follow-up, whichever occurred first. Any change in residence or survival status was traced by the residential registry in the study area annually. Loss to follow-up subjects was censored on the last date confirmed of their presence. Information on cause of death was confirmed by examining death certificates, which were provided by the Ministry of Health, Labour, and Welfare with permission from the Ministry of Internal Affairs and Communications^24^. The information of residency and death registration is requested by law in Japan.

### Assessment of exposure

All exposure data was collected from the baseline survey, which included a 44-item food self-administrated food frequency questionnaire (FFQ)^26^. The questions on measurements of green tea and coffee consumption were the same in Cohort I and Cohort II. Previously published JPHC Study papers were used for reference in determining categories of green tea and coffee consumption^27,28^. Green tea consumption was divided into five frequency categories: “rarely”, “ < 1 cup/day”, “1–2 cups/day”, “3–4 cups/day” and “ ≥ 5 cups/day”. Coffee consumption was also divided into five frequency categories: “rarely”, “1–2 days/week”, “3–4 days/week”, “1–2 cups/day” and “ ≥ 3 cups/day”. The validity of the FFQ was assessed using dietary records among 201 participants (94 men and 107 women) for 28 or 14 days^26^. In Cohort I, Spearman rank correlation coefficients between the FFQ and the dietary records were 0.57 for men and 0.63 for women for green tea consumption, whereas for coffee consumption the numbers were 0.42 for men and 0.38 for women^26^. In Cohort II, the coefficients were 0.37 for men and 0.43 for women for green tea consumption, while for coffee consumption the numbers were 0.59 for men and 0.51 for women^28^.

### Outcome ascertainment

The outcome of kidney cancer incidence as first cancer developed was defined according to the International Classification of Diseases for Oncology, Third Edition (ICD-O-3), including malignant neoplasm of kidney (C64). The sources of identification of cancer included active patient notifications made by major local hospitals in the study area, data linkage with population-based cancer registries, and death certificate information. Permission was obtained from relevant local governments.

### Statistical analysis

Hazard ratios (HRs) and 95% confidence intervals (95% CIs) for kidney cancer incidence were computed using cox proportional hazards regression models. Model 1 considered age (stratified by age groups: 45–49, 50–54, 55–59, 60–64, 65–69 and > 70 years old) and PHC area (10 areas) as covariates; and model 2 considered age, PHC area, smoking (past, current, never, missing), history of hypertension (yes, no, missing), history of diabetes (yes, no, missing), history of renal diseases (yes, no, missing), drinking frequency (rarely, 1–3 days/month, 1–2 days/week, 3–4 days/week, ≥ 5 days/week, missing), body mass index (< 18, 19–22, 23–25, 26–29, > 30, missing), physical activity (rarely, 1–3 days/month, 1–2 days/week, 3–4 days/week, almost daily, missing). Selection of confounding variables was mainly based on a previously published JPHC Study paper investigating the relationship between green tea and kidney cancer mortality^27^, as well as previous evidence about the relationship between kidney cancer and green tea and coffee intake^1,5–9^. Supplemental Fig. 1 of a directed acyclic graph (DAG) represents the relationship of variables and kidney cancer risk. The DAG was made via DAGitty version 3.0 with the model by Shrier and Platt^29,30^. Dummy variables were created to indicate missing information of confounding variables in data analysis. Complete case analysis was done in the sensitivity analysis as an alternative missing data method. Models were mutually controlled for green tea consumption and coffee consumption, and were created for both sexes separately and for the total population with sex added as a confounding variable. As the sex disparity in kidney cancer has been reported widely and was not likely to be fully explained by known risk factors^1,31^, analyses were done by stratifying sex in this study.

A sensitivity analysis was conducted by limiting kidney cancer cases only with subjective symptoms. Incidental detection of kidney cancer due to the widespread use of testing in addition to screening could lead to potential influence^32,33^. The detailed definition of subjective symptoms was not provided in this study. Another sensitivity analysis was performed by adjusting for additional variables, green vegetables intake and consumption of miso soup intake in Model 2 to check if the results changed. These two variables are considered as a proxy of healthy lifestyles in Japanese in relation to obesity and hypertension as shown in Supplemental Fig. 1^32,33^. Furthermore, data was right censored by omitting participants who were diagnosed with cancers other than kidney cancer in a sensitivity analysis to reduce potential bias. Stratification analyses were performed by stratifying smoking status (never versus past and current) and by body mass index (< 25 kg/m^2^ vs. ≥ 25 kg/m^2^) as previous evidence from the JPHC study has confirmed the increased risk of these two variables related to kidney cancer^34,35^. The biological mechanism underlying the carcinogenic effects of smoking is generally considered as its promotion to lipid peroxidation and oxygen free radicals’ formation, and that results in renal tubular damage^36^. Obesity plays a critical role in interlinked hormonal alterations, which could increase cancer incidence and progression^37^.

The cox proportional hazard assumption for all models was tested using scaled Schoenfeld residuals^38^. No violation was observed overall. P for trend was tested by treating the green tea and coffee category measures as continuous variables. All statistical analyses were conducted using Stata 16.1 (StataCorp, College Station, TX, USA), with a *P* value of less than 0.05 considered as statistically significant.

This study has been approved by the Institutional Review Board of the National Cancer Center Japan (approval number: 2015-085) and the Ethics Committee of Graduate School of Medicine, The University of Tokyo (approval number: 2019209NI). All methods were performed in accordance with relevant guidelines and regulations.

## Results

### Baseline characteristics

Baseline characteristics of participants according to green tea or coffee consumption are shown in Tables 1 and 2. A total of 286 cases of kidney cancer (199 in men, 0.4% in total population, 87 in women 0.2% in total population) were identified during 1,916,421 person-years (mean follow-up period, 19 years). The body mass index (BMI) among the total population were normal in all green tea and coffee consumption categories^39^. A growing trend in age was observed in participants with increased green tea intake. In contrast, a decreasing trend in age was observed in both men and women with increased coffee intake. Men and women who consumed a high intake of green tea (≥ 5 cups/day) seldom consumed black tea, oolong tea, or coffee. Participants with a high intake of coffee (≥ 3 cups/day) rarely consumed black tea or oolong tea.

**Table 1:** Baseline characteristics of JPHC Study participants according to green tea consumption (n = 102,463).

	Rarely	< 1 cup/day	1–2 cups/day	3–4 cups/day	≥ 5 cups/day
**Men (n = 48,647)**					
Participants	5705	6876	11,482	12,863	11,721
Incidence rate (per 100,000 person-time)	25.8	19.3	17.7	26.0	24.3
Age (y), mean (SD)	50.6 (7.57)	49.4 (7.29)	51.0 (7.98)	52.7 (8.19)	54.2 (7.80)
BMI (kg/m2), mean (SD)	23.9 (3.15)	23.8 (3.02)	23.5 (2.84)	23.3 (2.84)	23.4 (3.09)
Current smoker (%)	50.38	52.08	52.89	51.67	54.24
Current drinker (%)	74.83	80.321	81.19	78.17	73.82
History of hypertension (%)	16.16	15.21	17.42	19.09	17.73
History of diabetes (%)	6.36	6.41	6.34	6.61	6.86
History of renal diseases (%)	2.05	2.25	1.92	2.18	2.04
Sports or physical exercise almost daily (%)	5.63	4.74	4.72	5.04	5.41
Oolong tea intake < 1 cup/day (%)	79.83	77.54	81.58	85.27	86.96
Black tea intake < 1 cup/day (%)	95.71	93.98	94.35	94.43	95.14
Miso soup intake almost daily (%)	64.45	60.01	68.66	72.81	78.77
Green vegetables consumption almost daily (%)	24.19	17.50	20.25	22.79	28.16
Coffee consumption < 1 cup/day (%)	56.85	57.12	52.32	59.55	69.30
**Women (n = 53,816)**					
Participants	6,208	6,948	11,267	15,026	14,367
Incidence rate (per 100,000 person-time)	13.9	6.6	7.4	8.4	7.6
Age (y), mean (SD)	50.7 (7.44)	49.5 (7.34)	51.0 (8.02)	53.0 (8.28)	54.3 (7.92)
BMI (kg/m2), mean (SD)	23.6 (3.44)	23.5 (3.22)	23.3 (3.66)	23.3 (3.20)	23.5 (3.25)
Current smoker (%)	7.93	7.77	6.51	5.39	7.04
Current drinker (%)	19.76	27.01	25.84	23.33	22.61
History of hypertension (%)	15.34	13.60	14.41	16.32	17.30
History of diabetes (%)	3.38	2.53	2.70	2.73	3.50
History of renal diseases (%)	2.14	2.23	1.86	2.06	2.14
Sports or physical exercise almost daily (%)	4.77	3.90	4.54	4.71	5.13
Oolong tea intake < 1 cup/day (%)	76.12	73.84	78.51	85.09	85.37
Black tea intake < 1 cup/day (%)	95.27	93.06	91.70	91.89	93.19
Miso soup intake almost daily (%)	62.55	59.36	64.12	67.61	70.52
Green vegetables consumption almost daily (%)	28.64	27.36	28.27	30.31	33.83
Coffee consumption < 1 cup/day (%)	58.54	54.71	51.09	61.46	72.05

^a^ANOVA test for continuous variables or chi-square test for categorical variables; ^b^ D = standard deviation.

**Table 2:** Baseline characteristics of JPHC Study participants according to coffee consumption (n = 102,463).

	Rarely	1–2 days/week	3–4 days/week	1–2 cups/day	≥ 3 cups/day
**Men (n = 48,647)**					
Participants	14,630	8,666	5,665	12,735	6,951
Incidence rate (per 100,000 person-time)	27.8	20.1	19.2	22.8	18.0
Age (y), mean (SD)	54.1 (7.82)	52.9 (7.84)	51.7 (7.83)	50.8 (7.97)	48.6 (7.37)
BMI (kg/m2), mean (SD)	23.5 (2.94)	23.6 (2.90)	23.6 (3.35)	23.5 (2.85)	23.4 (3.01)
Current smoker (%)	43.33	48.33	51.72	55.94	71.21
Current drinker (%)	77.15	78.88	80.18	79.45	72.88
History of hypertension (%)	22.72	19.11	16.59	14.75	10.11
History of diabetes (%)	8.84	7.17	5.40	5.10	4.56
History of renal diseases (%)	2.39	2.04	2.17	1.81	1.91
Sports or physical exercise almost daily (%)	6.02	4.71	4.89	4.73	4.36
Oolong tea intake < 1 cup/day (%)	84.49	85.23	81.53	80.89	82.57
Black tea intake < 1 cup/day (%)	96.32	96.02	92.17	93.34	93.95
Miso soup intake almost daily (%)	76.53	74.73	69.74	66.15	60.96
Green vegetables consumption almost daily (%)	26.37	22.32	22.42	21.71	18.90
Green tea consumption < 1 cup/day (%)	24.61	23.65	26.86	25.38	31.34
**Women (n = 53,816)**					
Participants	17,132	9,854	5,792	15,724	5,314
Incidence rate (per 100,000 person-time)	13.0	4.1	8.8	6.3	7.0
Age (y), mean (SD)	55.3 (7.76)	53.2 (7.85)	51.5 (7.81)	50.0 (7.61)	47.5 (6.82)
BMI (kg/m2), mean (SD)	23.6 (3.74)	23.5 (3.18)	23.5 (3.12)	23.3 (3.14)	23.0 (3.07)
Current smoker (%)	4.40	4.17	4.77	7.58	17.93
Current drinker (%)	16.64	21.17	24.15	29.09	34.96
History of hypertension (%)	22.02	16.83	13.90	11.77	7.02
History of diabetes (%)	4.85	2.89	2.00	1.93	1.26
History of renal diseases (%)	2.43	2.08	2.05	1.70	1.99
Sports or physical exercise almost daily (%)	5.73	4.56	3.90	4.26	3.69
Oolong tea intake < 1 cup/day (%)	83.67	83.46	80.71	78.80	77.53
Black tea intake < 1 cup/day (%)	95.26	94.57	89.20	90.56	91.47
Miso soup intake almost daily (%)	72.71	71.46	66.02	59.91	52.30
Green vegetables consumption almost daily (%)	32.65	29.98	28.95	29.92	25.42
Green tea consumption < 1 cup/day (%)	23.13	20.69	24.74	24.96	33.80

^a^ANOVA test for continuous variables or chi-square test for categorical variables; ^b^SD = standard deviation.

### Green tea consumption and kidney cancer risk

**Figure 1:**
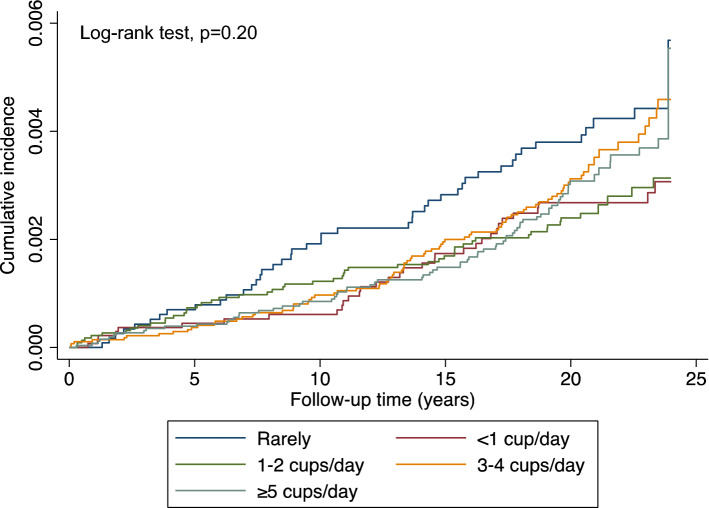
Kaplan–Meier survival curves of the cumulative incidence of kidney cancer among JPHC participants with different green tea consumption.

Figure 1 presents the association between the cumulative kidney cancer incidence rates and the frequency of green tea consumption using the Kaplan–Meier method. The cumulative incidence rates were not significantly different among the five groups with different green tea intake frequency (log-rank test, *p* = 0.20) (Fig. 1). For the total population, after adjusting for potential covariates, there was no statistically significant association between green tea intake and kidney cancer risk found with a p for trend of 0.39 in the multivariable model (Table 3). High green tea consumption, which refers to ≥ 5 cups/day in the study, showed a HR of 0.75 (95% CI: 0.51–1.12) in the total population when adjusted for multivariable, compared to the reference group, those rarely consuming green tea. The relationships were not statistically significant in men, with a multivariable-adjusted HR for the group consumed ≥ 5 cups/day of 0.96 (95% CI: 0.59–1.56). For women, a statistically significant inverse association was found between the highest frequency of green tea intake and kidney cancer risk, with the HR of women who had ≥ 5 cups/day green tea intake of 0.45 (95% CI: 0.23–0.89).

**Table 3:** Hazard ratio and 95% confidence interval for kidney cancer incidence according to green tea consumption in Japanese adults.

	Rarely	< 1 cup/day	1–2 cups/day	3–4 cups/day	≥ 5 cups/day	*P* for trend
**Total population (n = 102,463)**						
Person-years (n = 1,916,421)	227,248	260,360	419,765	517,512	491,536	
Number of cases (n = 286)	44	33	52	84	73	
Age and area adjusted HR	1.00 (reference)	0.71 (0.45–1.12)	0.71 (0.47–1.07)	0.90 (0.62–1.33)	0.73 (0.49–1.08)	0.45
Multivariable-adjusted HR	1.00 (reference)	0.73 (0.46–1.15)	0.74 (0.49–1.12)	0.94 (0.64–1.38)	0.75 (0.51–1.12)	0.39
**Men (n = 48,647)**						
Person-years (n = 877,150)	104,724	124,436	203,507	230,501	213,982	
Number of cases (n = 199)	27	24	36	60	52	
Age and area adjusted HR	1.00 (reference)	0.86 (0.49–1.50)	0.83 (0.50–1.39)	1.15 (0.72–1.85)	0.94 (0.58–1.53)	0.71
Multivariable-adjusted HR	1.00 (reference)	0.88 (0.50–1.53)	0.86 (0.51–1.44)	1.19 (0.74–1.92)	0.96 (0.59–1.56)	0.74
**Women (n = 53,816)**						
Person-years (n = 1,039,271)	122,524	135,924	216,258	287,011	277,554	
Number of cases(n = 87)	17	9	16	24	21	
Age and area adjusted HR	1.00 (reference)	0.47 (0.21–1.06)	0.52 (0.26–1.05)	0.54 (0.28–1.04)	0.42 (0.21–0.83)	0.05
Multivariable-adjusted HR	1.00 (reference)	0.49 (0.22–1.12)	0.57 (0.28–1.16)	0.59 (0.31–1.15)	0.45 (0.23–0.89)	0.05

^a^In multivariable-adjusted models for men and women, adjusted variables included age (stratified by age groups: 45–49, 50–54, 55–59, 60–64, 65–69 and > 70 years old), sex (men and women), PHC area (stratified by 10 areas), smoking (past, current, never, missing), history of hypertension (yes, no, missing), history of diabetes (yes, no, missing), history of renal diseases (yes, no, missing), drinking frequency (rarely, 1–3 days/month, 1–2 days/week, 3–4 days/week, ≥ 5 days/week, missing), body mass index (< 18, 19–22, 23–25, 26–29, > 30, missing), physical activity (rarely, 1–3 days/month, 1–2 days/week, 3–4 days/week, almost daily, missing). Models were mutually controlled coffee consumption. In the models for the total population, in addition to these variables, sex was also included.

### Coffee consumption and kidney cancer risk

**Figure 2:**
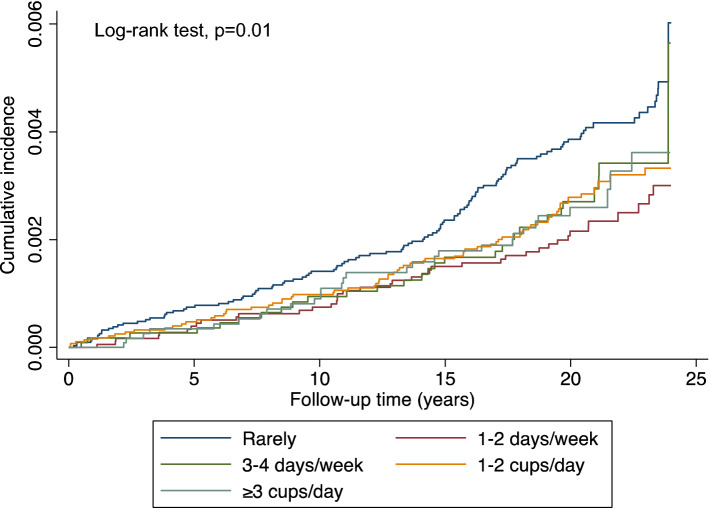
Kaplan–Meier survival curves of the cumulative incidence of kidney cancer among JPHC participants with different coffee consumption.

The association between the cumulative incidence rates of coffee consumption and kidney cancer is presented in Fig. 2. The cumulative kidney cancer incidence rates were significantly different among the groups with different coffee consumption frequency (log-rank test, *p* = 0.01) (Fig. 2). The associations related to kidney cancer risk were not statistically significant for the total population with a *p* trend of 0.36 (Table 4). No statistically significant results were observed in both men and women (Table 4). In the total population, individuals who consumed 1–2 cups of coffee per week had a multivariable-adjusted HR of 0.62 (95% CI: 0.43–0.89) compared to those who rarely consumed coffee. Women with the same coffee consumption frequency also had a significant difference (HR: 0.38, 95% CI: 0.18–0.81). No such relationship was found in men.

**Table 4:** Hazard ratio and 95% confidence interval for kidney cancer incidence according to coffee consumption in Japanese adults.

	Rarely	1–2 days/week	3–4 days/week	1–2 cups/day	≥ 3 cups/day	*P* for trend
**Total population (n = 102,463)**						
Person-years (n = 1,916,421)	594,335	352,760	217,595	529,994	221,737	
Number of cases (n = 286)	116	40	30	71	29	
Age and area adjusted HR	1.00 (reference)	0.61 (0.42–0.87)	0.79 (0.52–1.18)	0.87 (0.64–1.18)	0.84 (0.55–1.28)	0.46
Multivariable-adjusted HR	1.00 (reference)	0.62 (0.43–0.89)	0.80 (0.53–1.21)	0.89 (0.65–1.22)	0.84 (0.65–1.22)	0.36
**Men (n = 48,647)**						
Person-years (n = 877,150)	263,057	159,421	104,335	228,427	121,910	
Number of cases (n = 199)	73	32	20	52	22	
Age and area adjusted HR	1.00 (reference)	0.74 (0.49–1.13)	0.77 (0.47–1.26)	0.97 (0.67–1.40)	0.85 (0.52–1.39)	0.73
Multivariable-adjusted HR	1.00 (reference)	0.75 (0.49–1.14)	0.77 (0.47–1.27)	0.98 (0.68–1.43)	0.85 (0.51–1.40)	0.61
**Women (n = 53,816)**						
Person-years (n = 1,039,271)	331,278	193,339	113,260	301,567	99,827	
Number of cases(n = 87)	43	8	10	19	7	
Age and area adjusted HR	1.00 (reference)	0.36 (0.17–0.76)	0.85 (0.42–1.71)	0.68 (0.38–1.20)	0.86 (0.37–2.00)	0.39
Multivariable-adjusted HR	1.00 (reference)	0.38 (0.18–0.81)	0.92 (0.46–1.87)	0.70 (0.39–1.26)	0.84 (0.35–2.01)	0.31

^a^In multivariable-adjusted models for men and women, adjusted variables included age (stratified by age groups: 45–49, 50–54, 55–59, 60–64, 65–69 and > 70 years old), sex (men and women), PHC area (stratified by 10 areas), smoking (past, current, never, missing), history of hypertension (yes, no, missing), history of diabetes (yes, no, missing), history of renal diseases (yes, no, missing), drinking frequency (rarely, 1–3 days/month, 1–2 days/week, 3–4 days/week, ≥ 5 days/week, missing), body mass index (< 18, 19–22, 23–25, 26–29, > 30, missing), physical activity (rarely, 1–3 days/month, 1–2 days/week, 3–4 days/week, almost daily, missing). Models were mutually controlled coffee consumption. In the models for the total population, in addition to these variables, sex was also included.

### Sensitivity analysis

In the sensitivity analyses adjusting additional variables for green vegetables intake and consumption of miso soup, results were similar when compared to the multivariable-adjusted HRs. No significant associations were detected between green tea intake and kidney cancer in the total population or in men (Supplemental Table 1). In women, a consistent reduced risk was observed in the category of ≥ 5 cups green tea/day (HR: 0.45, 95% CI: 0.22–0.89; *p* trend = 0.06). Results for coffee consumption did not show statistically significant associations with kidney cancer. The reduced risks in the frequency of 1–2 days/week coffee intake were also found in total population and women, but not in men. In addition, no significant associations between kidney cancer risk and green tea and coffee intake were observed when limiting cases with subjective symptoms (Supplemental Table 2).

In the sensitivity analysis stratified by smoking status, no significant associations between green tea and kidney cancer risk were detected in the total population, in men or in women who never smoked (Supplemental Table 2). A significant inverse relationship between green tea intake and kidney cancer risk was found in women who ever smoked. (*p* = 0.04). In the sensitivity analysis stratified by BMI, no statistically significant relationships were observed for green tea and coffee consumption (Supplement Table 3).

Results in the complete case analysis were similar to the results using dummy variables (Supplemental Table 4). No statistically significant associations were established between both intakes of green tea and coffee and kidney cancer risk. When using censored data withdrawing participants diagnosed with other cancers, results showed no statistically significant associations between both intakes of green tea and coffee and kidney cancer risk but the same reduced risk in women had ≥ 5 cups green tea/day (Supplemental Table 5).

## Discussion

This is the first study, to the best of our knowledge, to evaluate the relationship between green tea intake and risk of kidney cancer development in a large population-based cohort study in Asia. In sum, we found no statistically significant associations between different levels of green tea intake and risk of kidney cancer in the total population. Nonetheless, there might be a decreasing trend between kidney cancer risk and higher green tea intake. The reduced risk shown in women in the highest level of green tea intake further indicated the potential association. For coffee consumption, no statistically significant relationships were established in the total population, in men or in women, which was not consistent with the meta-analysis published in 2022^16^.

Our study indicated a possible decreasing trend between high green tea intake and kidney cancer risk. A recent hospital-based case–control study that investigated the association between green tea intake and clear cell renal cell carcinoma (ccRCC) in a Chinese population proved the inhibitory effect of higher green tea intake (> 500 ml/day) against ccRCC^21^. The anticarcinogenic effect possibly credited to catechins and theaflavins in green tea is described in evidence from cell or animal studies^40–43^. The chemo-preventative action of ( −)-epigallocatechin-3-gallate (EGCG), a type of catechin founded in green tea, has been proven through preservation of gap junction intercellular communication (GJIC) of renal epithelial cells when treated with a renal carcinogen in vitro^42,43^. This kind of restoration of GJIC indicated the distinguishing feature that green tea has when targeting renal cell tumors. The present study indicated such preventive effects of green tea seemed to exist, especially for women who consume a great amount of green tea. When stratified by smoking status, a statistically significant relationship was found between higher green tea intake and kidney cancer risk in ever-smoked women (*p* trend = 0.04). The multivariable-adjusted HRs in the group of ≥ 5 cups green tea/day of ever-smoked women (0.06, 95% CI: 0.004–0.79) and never-smoked women (0.57, 95% CI: 0.27–1.18) were similar, indicating there was hardly any difference between women with different smoking statuses. Still, there might be undiscovered effects in ever smoked women, considering the larger HR and the upper limit of 1.18 in the 95% confidence interval in never-smoked women. In the sensitivity analyses stratified by BMI, the HRs in the group of ≥ 5 cups green tea/day of women with BMI < 25 (0.50, 95% CI: 0.28–1.19) and women with BMI ≥ 25 (0.44, 95% CI: 0.14–1.33) were very close. In addition, the results in stratified BMI analysis were similar to the main results, suggesting the same decreasing trend between higher intakes of green tea and kidney cancer risk in women. The insignificant relationship is likely due to the small number of cases in women.

The findings suggest a sex difference in the association between green tea consumption and kidney cancer risk. One possible mechanism could be the different action of sex hormones in women who regularly drank green tea, in particularly related to estrogen metabolism^44,45^. Estrogen could prevent renal cell carcinoma cell progression by activating estrogen receptor-β^46^. Studies have shown that intake of green tea was associated with estrogen circulation and metabolism^47,48^. Further investigation is warranted to discover latent risk factors and mechanisms that may cause the sex discrepancy of the association between green tea intake and kidney cancer risk.

The null relationship between coffee intake and kidney cancer risk was not consistent with the recent meta-analysis by Rhee et al. in 2022^16^. A significant inverse relationship between the highest versus lowest coffee intake and renal cancer risk was established in their meta-analysis of ten identified cohort studies, with a summary relative risk (RR) of 0.88 (95% CI: 0.78–0.99)^16^. Among the ten cohort studies, four were conducted in the region of North America^16^. And the summary RR of other regions was 0.93 (95% CI: 0.72–1.21), indicating the difference between geographical regions^16^. Our results were consistent with results from another cohort study in Japan, the Japan Collaborative Cohort Study (JACC study)^49^, which was included in the meta-analysis by Rhee et al. The JACC study reported an age-and sex-adjusted HR of 2.69 (95% CI: 0.89–8.10) among participants who drank more than three cups of coffee per day, compared with participants with no coffee intake^49^. The heterogeneity in results among different studies included in the meta-analysis on coffee may be ascribable to methodological errors. Our results have shown a statistically significant reduced risk in the frequency of 1–2 cups of coffee per week with a HR of 0.62 (95CI%: 0.43–0.89) in the total population. The small number of cases could mask the true effect of a potential inverse relationship between coffee intake and kidney cancer.

The foremost strength of our study was its large-scale prospective design including several public health center areas over Japan, along with a low proportion of loss to follow-up (0.4%). Therefore, the potential of recall bias and selection bias was largely reduced. Furthermore, the data from the comprehensive FFQ, and evaluation from a credible registry system enhanced the accuracy of results. This study has some limitations. First, the main limitation of the study is the low statistical power. The small number of cases and wide CIs due to the small number of cases indicated uncertainty of the results and bias the true effects. The inconsistent results observed in the complete-case analysis also suggested that missing values led to reduced statistical power. Second, the results could be biased due to limitations of FFQ assessment. Changes in dietary patterns were not reflected since only baseline data was used in the analysis. The National Health and Nutrition Survey in Japan^50^, suggests green tea and coffee consumption remained stable between 2011 and 2018 and it is likely that the trends haven’t changed for decades. Variations of other lifestyle patterns may exist during the follow-up years, which could lead to potential misclassification. The effect of different methods applied in drinking green tea and coffee was not considered due to a lack of questions about brewing process in beverage consumption in the FFQ. Third, residual confounding effects may exist due to the inability to adjust all potential risk factors for kidney cancer based on limited evidence. Possible underlying risk factors include influence of sex hormones^51,52^ and occupational exposure to heavy metals, gasoline, or diesel engine exhausts^53,54^. Forth, although the mean follow-up period was long up to 19 years in this study, the small number of kidney cancers detected in each exposure category could lead to uncertainty. Lastly, the moderate correlations between the FFQ and the dietary records of green tea and coffee consumption indicated moderate reproducibility, which could lead to error in estimates when quantifying actual green tea and coffee intake.

In conclusion, our findings suggest that green tea, but not coffee consumption may be inversely related to kidney cancer incidence in Japanese adults, with a significant association observed in Japanese women.

## Supplementary Information

Supplementary Information.

## Data Availability

For information on how to submit an application for gaining access to JPHC data please follow the instructions at http://epi.ncc.go.jp/en/jphc/805/8155.html.
