# Supplementary material for: Green tea and coffee consumption and risk of kidney cancer in Japanese adults
Source: Sci Rep. 2022 Nov 24;12:20274. doi: 10.1038/s41598-022-24090-z (PMC9700732; doi:10.1038/s41598-022-24090-z)
Supplement: Supplementary file 1 — Supplementary Information. [file 41598_2022_24090_MOESM1_ESM.pdf]

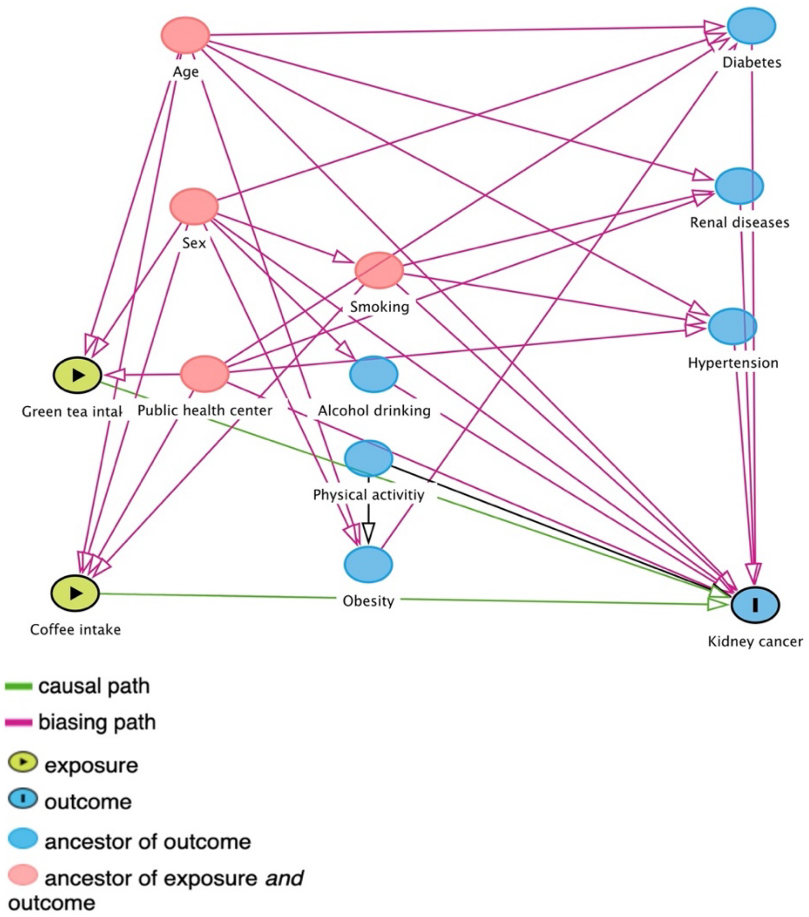

Supplemental Figure 1. Directed cyclic graph showing the relationship between variables and kidney cancer risk[1] Based on DAGitty version 3.0 (Johannes Textor, Nijmegen, the Netherlands)[2].

Supplemental Table 1. Hazard ratio (95% confidence interval) for the associations between kidney cancer risk and green tea and coffee consumption in JPHC participants adjusting for additional variables

**Green tea consumption**	**Rarely**	**<1 cup/day**	**1-2 cups/day**	**3-4 cups/day**	**≥5 cups/day**	**P for trend**
Total population (n=102,463)						
Person-years (n=1,916,421)	227,248	260,360	419,765	517,512	491,536	
Number of cases (n=286)	44	33	52	84	73	
Multivariable-adjusted HR	1.00 (reference)	0.73 (0.46-1.15)	0.74 (0.49-1.12)	0.94 (0.64-1.38)	0.75 (0.51-1.12)	0.43
Men (n=48,646)						
Person-years (n=877,150)	104,724	124,436	203,507	230,501	213,982	
Number of cases (n=199)	27	24	36	60	52	
Multivariable-adjusted HR	1.00 (reference)	0.92 (0.53-1.61)	0.88 (0.53-1.48)	1.22 (0.76-1.98)	0.96 (0.59-1.57)	0.81
Women (n=53,816)						
Person-years (n=1,039,271)	122,524	135,924	216,258	287,011	277,554	
Number of cases(n=87)	17	9	16	24	21	
Multivariable-adjusted HR	1.00 (reference)	0.49 (0.21-1.11)	0.57 (0.28-1.15)	0.59 (0.30-1.15)	0.45 (0.22-0.89)	0.06

**Coffee consumption**	**Rarely**	**1-2 days/week**	**3-4 days/week**	**1-2 cups/day**	**≥3 cups/day**	
Total population (n=102,463)						
Person-years (n=1,916,421)	594,335	352,760	217,595	529,994	221,737	
Number of cases (n=286)	116	40	30	71	29	
Multivariable-adjusted HR	1.00 (reference)	0.62 (0.43-0.89)	0.80 (0.5131.21)	0.89 (0.65-1.22)	0.84 (0.55-1.30)	0.36
Men (n=48,643)						

Person-years (n=877,150)	263,057	159,421	104,335	228,427	121,910	
Number of cases (n=199)	73	32	20	52	22	
Multivariable-adjusted HR	1.00 (reference)	0.76 (0.50-1.16)	0.79 (0.48-1.31)	0.99 (0.69-1.44)	0.85 (0.51-1.41)	0.59
Women (n=53,816)						
Person-years (n=1,039,271)	331,278	193,339	113,260	301,567	99,827	
Number of cases(n=87)	43	8	10	19	7	
Multivariable-adjusted HR	1.00 (reference)	0.37 (0.17-0.80)	0.90 (0.45-1.82)	0.70 (0.39-1.26)	0.84 (0.35-2.01)	0.31

a In multivariable-adjusted models for men and women, adjusted variables included age (stratified by age groups: 45-49, 50-54, 55-59, 60-64, 65-69 and >70 years old), sex (men and women), PHC area (stratified by 10 areas), smoking (past, current, never, missing), history of hypertension (yes, no, missing), history of diabetes (yes, no, missing), history of renal diseases (yes, no, missing), drinking frequency (rarely, 1-3 days/month, 1-2 days/week, 3-4 days/week, ≥5 days/week, missing), body mass index (<18, 19-22, 23-25, 26-29, >30, missing), and physical activity (rarely, 1-3 days/month, 1-2 days/week, 3-4 days/week, almost daily, missing), green vegetables intake (rarely, 1-2 days/week, 3-4 days/week, almost daily, missing) and consumption of miso soup (rarely, <1 day/week, <1 time/day, almost daily, missing). Models were mutually controlled coffee consumption. In the models for the total population, in addition to these variables, sex was also included.

Supplemental Table 2. Hazard ratio (95% confidence interval) for the associations between kidney cancer risk and green tea and coffee consumption in JPHC participants stratified by smoking status

**Green tea consumption**	**Rarely**	**<1 cup/day**	**1-2 cups/day**	**3-4 cups/day**	**≥5 cups/day**	**P for trend**
Total population (n=102,463)						
Never smokers (n=60,891)	7,165	8,095	13,084	17,012	15,535	
Number of cases (n=128)	25	14	23	37	29	
Multivariable-adjusted HR	1.00 (reference)	0.58 (0.30-1.13)	0.69 (0.38-1.24)	0.85 (0.49-1.46)	0.65 (0.36-1.15)	0.33
Past and current smokers (n=41,572)	4,748	5,729	9,665	10,877	10,553	
Number of cases (n=158)	19	19	29	47	44	
Multivariable-adjusted HR	1.00 (reference)	0.92 (0.49-1.76)	0.85 (0.47-1.55)	1.12 (0.64-1.97)	0.93 (0.53-1.65)	0.08
Men (n=48,647)						
Never smokers (n=11,522)	1,585	1,803	2,724	2,990	2,420	
Number of cases (n=51)	11	5	11	15	9	
Multivariable-adjusted HR	1.00 (reference)	0.52 (0.18-1.53)	0.94 (0.39-2.27)	1.13 (0.49-2.57)	0.74 (0.29-2.86)	0.96
Past and current smokers (m=37,125)	4,120	5,073	8,758	9,873	9,301	
Number of cases (n=148)	16	19	25	45	43	
Multivariable-adjusted HR	1.00 (reference)	1.11 (0.57-2.17)	0.89 (0.46-1.70)	1.31 (0.72-2.38)	1.12 (0.61-2.05)	0.53
Women (n=53,816)						
Never smokers (n=49,369)	5,580	6,292	10,360	14,022	13,115	
Number of cases (n=77)	14	9	12	22	20	
Multivariable-adjusted HR	1.00 (reference)	0.61 (0.26-1.42)	0.55 (0.25-1.21)	0.70 (0.34-1.42)	0.57 (0.27-1.18)	0.20

Past and current smokers (n=4,447)	628	656	907	1,004	1,252	
Number of cases (n=10)	3	0	4	2	1	
Multivariable-adjusted HR	1.00 (reference)	/	0.43 (0.07-2.60)	0.19 (0.01-1.11)	0.06 (0.004-0.79)	0.04

**Coffee consumption**	**Rarely**	**1-2 days/week**	**3-4 days/week**	**1-2 cups/day**	**≥3 cups/day**	**P for trend**
Total population (n=102,463)						
Never smokers (n=60,891)	20,336	11,549	6,815	17,059	5,132	
Number of cases (n=128)	64	13	14	25	12	
Multivariable-adjusted HR	1.00 (reference)	0.40 (0.22-0.73)	0.81 (0.45-1.46)	0.65 (0.40-1.06)	1.25 (0.65-2.39)	0.34
Past and current smokers (n=41,572)	11,426	6,971	4,642	11,400	7,133	
Number of cases (n=158)	52	27	16	46	17	
Multivariable-adjusted HR	1.00 (reference)	0.85 (0.53-1.35)	0.83 (0.47-1.47)	1.16 (0.76-1.75)	0.76 (0.43-1.35)	0.80
Men (n=48,647)						
Never smokers (n=11,522)	4,193	2,259	1,380	2,788	902	
Number of cases (n=51)	24	6	6	9	6	
Multivariable-adjusted HR	1.00 (reference)	0.49 (0.20-1.20)	0.80 (0.32-2.00)	0.54 (0.25-1.18)	1.42 (0.56-3.60)	0.58
Past and current smokers (m=37,125)	10,437	6,407	4,285	9,947	6,049	
Number of cases (n=148)	49	26	14	43	16	
Multivariable-adjusted HR	1.00 (reference)	0.87 (0.54-1.40)	0.77 (0.42-1.40)	1.18 (0.77-1.81)	0.80 (0.44-1.44)	0.91
Women (n=53,816)						
Never smokers (n=49,369)	16,143	9,290	5,435	14,271	4,230	

Number of cases (n=77)	40	7	8	16	6	
Multivariable-adjusted HR	1.00 (reference)	0.36 (0.16-0.81)	0.81 (0.37-1.76)	0.72 (0.38-1.34)	1.12 (0.45-2.80)	0.40
Past and current smokers (n=4,447)	989	564	357	1,453	1,084	
Number of cases (n=10)	3	1	2	3	1	
Multivariable-adjusted HR	1.00 (reference)	0.38 (0.04-5.08)	1.37 (1.17-11.07)	0.57 (0.07-3.98)	0.43 (0.03-5.90)	0.55

a In multivariable-adjusted models for men and women, adjusted variables included age (stratified by age groups: 45-49, 50-54, 55-59, 60-64, 65-69 and >70 years old), sex (men and women), PHC area (stratified by 10 areas), smoking (past, current, never, missing), history of hypertension (yes, no, missing), history of diabetes (yes, no, missing), history of renal diseases (yes, no, missing), drinking frequency (rarely, 1-3 days/month, 1-2 days/week, 3-4 days/week, ≥5 days/week, missing), body mass index (<18, 19-22, 23-25, 26-29, >30, missing), and physical activity (rarely, 1-3 days/month, 1-2 days/week, 3-4 days/week, almost daily, missing). Models were mutually controlled coffee consumption. In the models for the total population, in addition to these variables, sex was also included.

Supplemental Table 3. Hazard ratio (95% confidence interval) for the associations between kidney cancer risk and green tea and coffee consumption in JPHC participants stratified by body mass index (BMI)

Green tea consumption	Rarely	<1 cup/day	1-2 cups/day	3-4 cups/day	≥5 cups/day	P for trend
Total population (n=102,463)						
BMI<25 (n=74,479)	8,188	9,743	16,835	20,756	18,956	
Number of cases (n=178)	25	21	37	47	48	
Multivariable-adjusted HR	1.00 (reference)	0.81 (0.45-1.46)	0.82 (0.48-1.38)	0.80 (0.48-1.34)	0.78 (0.47-1.30)	0.35
BMI≥25 (n=27,984)	3,725	4,081	5,914	7,133	7,131	
Number of cases (n=108)	19	12	15	37	25	
Multivariable-adjusted HR	1.00 (reference)	0.63 (0.30-1.31)	0.57 (0.29-1.15)	1.20 (0.67-2.16)	0.72 (0.38-1.34)	0.93
Men (n=48,647)						
BMI<25 (n=35,426)	3,866	4,733	8,418	9,655	8,754	
Number of cases (n=126)	15	15	25	37	34	
Multivariable-adjusted HR	1.00 (reference)	0.99 (0.48-2.05)	0.94 (0.48-1.82)	1.12 (0.60-2.10)	0.98 (0.52-1.85)	0.97
BMI≥25 (n=13,221)	1,839	2,143	3,064	3,208	2,967	
Number of cases (n=73)	12	9	11	23	18	
Multivariable-adjusted HR	1.00 (reference)	0.75 (0.31-1.80)	0.71 (0.31-1.65)	1.34 (0.64-2.78)	0.90 (0.42-1.93)	0.70
Women (n=53,816)						
BMI<25 (n=39,053)	4,322	5,010	8,417	11,101	10,203	
Number of cases (n=52)	10	6	12	10	14	
Multivariable-adjusted HR	1.00 (reference)	0.54 (0.19-1.50)	0.67 (0.28-1.58)	0.40 (0.58-0.99)	0.50 (0.28-1.19)	0.07

BMI≥25 (n=14,763)	1,886	1,938	2,850	3,925	4,164	
Number of cases (n=35)	7	3	4	14	7	
Multivariable-adjusted HR	1.00 (reference)	0.43 (0.11-1.70)	0.48 (0.11-1.38)	0.97 (0.37-2.58)	0.44 (0.14-1.33)	0.44
**Coffee consumption**	**Rarely**	**1-2 days/week**	**3-4 days/week**	**1-2 cups/day**	**≥3 cups/day**	**P for trend**
Total population (n=102,463)						
BMI<25 (n=74,479)	22,602	13,275	8,266	21,094	9,241	
Number of cases (n=178)	73	26	17	41	21	
Multivariable-adjusted HR	1.00 (reference)	0.64 (0.41-1.01)	0.72 (0.42-1.24)	0.80 (0.54-1.20)	0.99 (0.58-1.67)	0.42
BMI≥25 (n=27,984)	9,159	5,245	3,191	7,365	3,024	
Number of cases (n=108)	43	14	13	30	8	
Multivariable-adjusted HR	1.00 (reference)	0.59 (0.32-1.08)	0.93 (0.49-1.75)	1.05 (0.64-1.72)	0.61 (0.28-1.34)	0.59
Men (n=48,647)						
BMI<25 (n=35,426)	10,666	6,237	4,058	9,342	5,123	
Number of cases (n=126)	47	22	11	32	14	
Multivariable-adjusted HR	1.00 (reference)	0.81 (0.49-1.35)	0.68 (0.35-1.31)	0.95 (0.59-1.53)	0.89 (0.47-1.68)	0.57
BMI≥25 (n=13,221)	3,964	2,429	1,607	3,393	1,828	
Number of cases (n=73)	26	10	9	20	8	
Multivariable-adjusted HR	1.00 (reference)	0.66 (0.32-1.38)	0.94 (0.44-2.04)	1.04 (0.56-1.90)	0.76 (0.33-1.75)	0.91
Women (n=53,816)						
BMI<25 (n=39,053)	11,937	7,038	4,208	11,752	4,118	

Number of cases (n=52)	26	4	6	9	7	
Multivariable-adjusted HR	1.00 (reference)	0.31 (0.11-0.89)	0.88 (0.35-2.17)	0.51 (0.23-1.17)	1.28 (0.49-3.30)	0.46
BMI≥25 (n=14,763)	5,195	2,816	1,584	3,972	1,196	
Number of cases (n=35)	17	4	4	10	0	
Multivariable-adjusted HR	1.00 (reference)	0.45 (0.14-1.39)	0.95 (0.231-2.90)	1.01 (0.43-2.41)	/	0.39

a In multivariable-adjusted models for men and women, adjusted variables included age (stratified by age groups: 45-49, 50-54, 55-59, 60-64, 65-69 and >70 years old), sex (men and women), PHC area (stratified by 10 areas), smoking (past, current, never, missing), history of hypertension (yes, no, missing), history of diabetes (yes, no, missing), history of renal diseases (yes, no, missing), drinking frequency (rarely, 1-3 days/month, 1-2 days/week, 3-4 days/week, ≥5 days/week, missing), body mass index (<18, 19-22, 23-25, 26-29, >30, missing), and physical activity (rarely, 1-3 days/month, 1-2 days/week, 3-4 days/week, almost daily, missing). Models were mutually controlled coffee consumption. In the models for the total population, in addition to these variables, sex was also included.

Supplemental Table 4. Hazard ratio (95% confidence interval) for the associations between kidney cancer risk and green tea and coffee consumption in JPHC participants analyzed using complete case data

**Green tea consumption**	**Rarely**	**<1 cup/day**	**1-2 cups/day**	**3-4 cups/day**	**≥5 cups/day**	**P for trend**
Total population (n=63,479)						
Person-years (n=1,226,132)	170,431	184,122	250,687	300,277	316,615	
Number of cases (n=210)	34	27	37	61	51	
Age and area adjusted HR	1.00 (reference)	0.76 (0.46-1.27)	0.76 (0.47-1.22)	1.00 (0.65-1.56)	0.74 (0.47-1.17)	0.54
Multivariable-adjusted HR	1.00 (reference)	0.81 (0.48-1.35)	0.80 (0.49-1.29)	1.05 (0.68-1.64)	0.77 (0.48-1.21)	0.55
Men (n=31,717)						
Person-years (n=588,566)	77,836	89,995	126,905	142,220	147,610	
Number of cases (n=146)	23	20	26	40	37	
Age and area adjusted HR	1.00 (reference)	0.86 (0.47-1.58)	0.81 (0.46-1.45)	1.05 (0.61-1.80)	0.86 (0.50-1.48)	0.87
Multivariable-adjusted HR	1.00 (reference)	0.93 (0.51-1.71)	0.87 (0.49-1.55)	1.11 (0.64-1.91)	0.87 (0.50-1.51)	0.87
Women (n=31,762)						
Person-years (n=637,566)	92,595	94,127	123,782	158,057	169,004	
Number of cases(n=64)	11	7	11	21	14	
Age and area adjusted HR	1.00 (reference)	0.57 (0.22-1.47)	0.63 (0.27-1.49)	0.89 (0.41-1.92)	0.53 (0.23-1.21)	0.38
Multivariable-adjusted HR	1.00 (reference)	0.60 (0.23-1.57)	0.69 (0.29-1.64)	0.96 (0.44-2.08)	0.55 (0.23-1.27)	0.39

**Coffee consumption**	**Rarely**	**1-2 days/week**	**3-4 days/week**	**1-2 cups/day**	**≥3 cups/day**
Total population (n=63,479)					
Person-years (n=1,226,132)	408,030	232,475	145,275	318,694	121,959

Number of cases (n=210)	78	33	22	54	23	
Age and area adjusted HR	1.00 (reference)	0.76 (0.51-1.14)	0.85 (0.53-1.37)	1.02 (0.71-1.46)	1.11 (0.69-1.80)	0.63
Multivariable-adjusted HR	1.00 (reference)	0.79 (0.53-1.20)	0.89 (0.55-1.44)	1.06 (0.74-1.53)	1.16 (0.71-1.91)	0.49
Men (n=31,717)					.	
Person-years (n=588,566)	188,827	110,789	73,393	144,451	71,406	
Number of cases (n=146)	48	27	16	38	17	
Age and area adjusted HR	1.00 (reference)	0.99 (0.62-1.59)	0.94 (0.53-1.65)	1.17 (0.76-1.80)	1.17 (0.66-2.06)	0.45
Multivariable-adjusted HR	1.00 (reference)	1.03 (0.65-1.66)	0.98 (0.55-1.73)	1.19 (0.77-1.86)	1.19 (0.66-2.13)	0.44
Women (n=31,762)						
Person-years (n=637,566)	219,203	121,686	71,882	174,243	50,553	
Number of cases(n=64)	30	6	6	16	6	
Age and area adjusted HR	1.00 (reference)	0.37 (0.16-0.90)	0.69 (0.29-1.69)	0.77 (0.40-1.46)	1.07 (0.42-2.70)	0.77
Multivariable-adjusted HR	1.00 (reference)	0.39 (0.16-0.95)	0.75 (0.31-1.85)	0.80 (0.42-1.54)	1.20 (0.46-3.09)	0.82

a In the models for the total population, sex was also included; b In multivariable-adjusted models for men and women, adjusted variables included age (stratified by age groups: 45-49, 50-54, 55-59, 60-64, 65-69 and >70 years old), sex (men and women), PHC area (stratified by 10 areas), smoking (past, current, never, missing), history of hypertension (yes, no, missing), history of diabetes (yes, no, missing), history of renal diseases (yes, no, missing), drinking frequency (rarely, 1-3 days/month, 1-2 days/week, 3-4 days/week, ≥5 days/week, missing), body mass index (<18, 19-22, 23-25, 26-29, >30, missing), and physical activity (rarely, 1-3 days/month, 1-2 days/week, 3-4 days/week, almost daily, missing). Models were mutually controlled coffee consumption. In the models for the total population, in addition to these variables, sex was also included.

Supplementary Table 5. Hazard ratio (95% confidence interval) for the associations between kidney cancer risk and green tea and coffee consumption in JPHC participants censoring participants diagnosed with other cancers

**Green tea consumption**	**Rarely**	**<1 cup/day**	**1-2 cups/day**	**3-4 cups/day**	**≥5 cups/day**	**P for trend**
Total population (n=102,125)						
Person-years (n=1,845,590)	219,506	251,829	405,095	497,221	471,939	
Number of cases (n=268)	42	32	47	79	68	
Age and area adjusted HR	1.00 (reference)	0.72 (0.46-1.15)	0.68 (0.44-1.04)	0.91 (0.62-1.35)	0.72 (0.48-1.09)	0.45
Multivariable-adjusted HR	1.00 (reference)	0.76 (0.49-1.22)	0.72 (0.47-1.10)	0.96 (0.65-1.43)	0.75 (0.50-1.13)	0.42
Men (n=46,390)						
Person-years (n=838,358)	100,660	119,959	195,007	219,613	203,119	
Number of cases (n=186)	27	23	31	56	49	
Age and area adjusted HR	1.00 (reference)	0.83 (0.47-1.45)	0.72 (0.43-1.22)	1.10 (0.68-1.78)	0.90 (0.55-1.47)	0.78
Multivariable-adjusted HR	1.00 (reference)	0.89 (0.50-1.56)	0.77 (0.45-1.31)	1.17 (0.72-1.90)	0.92 (0.56-1.51)	0.87
Women (n=55,735)						
Person-years (n=1,007,232)	118,846	131,870	210,088	277,608	268,820	
Number of cases(n=82)	15	9	16	23	19	
Age and area adjusted HR	1.00 (reference)	0.54 (0.23-1.24)	0.60 (0.29-1.24)	0.60 (0.30-1.20)	0.44 (0.21-0.91)	0.07
Multivariable-adjusted HR	1.00 (reference)	0.57 (0.25-1.31)	0.66 (0.32-1.38)	0.67 (0.33-1.33)	0.47 (0.23-0.97)	0.08

**Coffee consumption**	**Rarely**	**1-2 days/week**	**3-4 days/week**	**1-2 cups/day**	**≥3 cups/day**
Total population (n=102,125)					

Person-years (n=1,845,590)	570,373	338,627	209,912	511,664	215,014	
Number of cases (n=268)	107	37	28	68	28	
Age and area adjusted HR	1.00 (reference)	0.62 (0.42-0.90)	0.80 (0.52-1.21)	0.91 (0.66-1.25)	0.89 (0.58-1.37)	0.72
Multivariable-adjusted HR	1.00 (reference)	0.64 (0.44-0.93)	0.83 (0.54-1.26)	0.90 (0.58-1.40)	0.73 (0.52-1.03)	0.59
Men (n=46,390)						
Person-years (n=838,358)	250,161	151,643	99,992	219,034	117,528	
Number of cases (n=186)	67	31	18	49	21	
Age and area adjusted HR	1.00 (reference)	1.45 (0.71-2.97)	0.48 (0.14-1.67)	0.50 (0.20-1.25)	0.43 (0.12-1.53)	0.74
Multivariable-adjusted HR	1.00 (reference)	0.82 (0.53-1.25)	0.79 (0.70-1.52)	1.03 (0.70-1.52)	0.90 (0.53-1.50)	0.70
Women (n=55,735)						
Person-years (n=1,007,232)	320,212	186,984	109,920	292,630	97,486	
Number of cases(n=82)	40	6	10	19	7	
Age and area adjusted HR	1.00 (reference)	0.23 (0.03-1.84)	1.88 (0.55-6.43)	0.84 (0.23-2.99)	2.18 (0.52-9.13)	0.41
Multivariable-adjusted HR	1.00 (reference)	0.30 (0.13-0.72)	0.98 (0.48-1.99)	0.77 (0.42-1.40)	0.93 (0.39-2.26)	0.59

a In the models for the total population, sex was also included; b In multivariable-adjusted models for men and women, adjusted variables included age (stratified by age groups: 45-49, 50-54, 55-59, 60-64, 65-69 and >70 years old), sex (men and women), PHC area (stratified by 10 areas), smoking (past, current, never, missing), history of hypertension (yes, no, missing), history of diabetes (yes, no, missing), history of renal diseases (yes, no, missing), drinking frequency (rarely, 1-3 days/month, 1-2 days/week, 3-4 days/week, ≥5 days/week, missing), body mass index (<18, 19-22, 23-25, 26-29, >30, missing), and physical activity (rarely, 1-3 days/month, 1-2 days/week, 3-4 days/week, almost daily, missing). Models were mutually controlled coffee consumption. In the models for the total population, in addition to these variables, sex was also included.

Supplemental Reference:

1. Shrier, I, Platt, W. Reducing bias through directed acyclic graphs. *BMC medical research methodology*, **8.1**, 1-15. (2008)
2. Textor J. Drawing and analyzing causal DAGs with DAGitty. *arXiv preprint arXiv*,1508.04633. (2015)
